# Variation in targetable genomic alterations in non-small cell lung cancer by genetic ancestry, sex, smoking history, and histology

**DOI:** 10.1186/s13073-022-01041-x

**Published:** 2022-04-15

**Authors:** Elio Adib, Amin H. Nassar, Sarah Abou Alaiwi, Stefan Groha, Elie W. Akl, Lynette M. Sholl, Kesi S. Michael, Mark M. Awad, Pasi A. Jӓnne, Alexander Gusev, David J. Kwiatkowski

**Affiliations:** 1 Department of Medicine - Pulmonary Division, Brigham and Women’s Hospital, Harvard Medical School, 20 Shattuck St, Boston, MA 02215 USA; 2 Lank Center for Genitourinary Oncology, Dana-Farber Cancer Institute, Boston, MA USA; 3 Department of Medical Oncology, Dana-Farber Cancer Institute, Boston, MA USA; 4 Division of Genetics, Department of Medicine, Brigham and Women’s Hospital and Harvard Medical School, Boston, MA USA; 5 Department of Pathology, Brigham and Women’s Hospital, Harvard Medical School, Boston, MA USA; 6 Lowe Center for Thoracic Oncology, Dana-Farber Cancer Institute, Boston, MA USA

**Keywords:** Non-small cell lung cancer, Targeted therapies, Targetable genomic alterations, Genetic ancestry, Smoking

## Abstract

**Background:**

Genomic alterations in 8 genes are now the targets of FDA-approved therapeutics in non-small cell lung cancer (NSCLC), but their distribution according to genetic ancestry, sex, histology, and smoking is not well established.

**Methods:**

Using multi-institutional genetic testing data from GENIE, we characterize the distribution of targetable genomic alterations in 8 genes among 8675 patients with NSCLC (discovery cohort: DFCI, *N* = 3115; validation cohort: Duke, Memorial Sloan Kettering Cancer Center, Vanderbilt, *N* = 5560). For the discovery cohort, we impute genetic ancestry from tumor-only sequencing and identify differences in the frequency of targetable alterations across ancestral groups, smoking pack-years, and histologic subtypes.

**Results:**

We identified variation in the prevalence of *KRAS*^*G12C*^, sensitizing *EGFR* mutations, *MET* alterations, *ALK*, and *ROS1* fusions according to the number of smoking pack-years. A novel method for computing continental (African, Asian, European) and Ashkenazi Jewish ancestries from panel sequencing enables quantitative analysis of the correlation between ancestry and mutation rates. This analysis identifies a correlation between Asian ancestry and *EGFR* mutations and an anti-correlation between Asian ancestry and *KRAS*^*G12C*^ mutation. It uncovers 2.7-fold enrichment for *MET* exon 14 skipping mutations and amplifications in patients of Ashkenazi Jewish ancestry. Among never/light smokers, targetable alterations in LUAD are significantly enriched in those with Asian (80%) versus African (49%) and European (55%) ancestry. Finally, we show that 5% of patients with squamous cell carcinoma (LUSC) and 17% of patients with large cell carcinoma (LCLC) harbor targetable alterations.

**Conclusions:**

Among patients with NSCLC, there was significant variability in the prevalence of targetable genomic alterations according to genetic ancestry, histology, and smoking. Patients with LUSC and LCLC have 5% rates of targetable alterations supporting consideration for sequencing in those subtypes.

**Supplementary Information:**

The online version contains supplementary material available at 10.1186/s13073-022-01041-x.

## Background

Non-small cell lung cancer (NSCLC) is the most common cause of cancer-related death in the USA and around the world [1]. In the last two decades, recognition of an increasing number of specific mutations and corresponding molecular-targeted therapeutics has completely changed the clinical approach to the care of lung adenocarcinoma (LUAD) [2].

To date, there are eight genes that constitute well-defined targets of Food and Drug Administration (FDA)-approved drugs: *EGFR*, *KRAS*, *ALK*, *MET*, *ROS1*, *BRAF*, *RET*, and *NTRK.* While the distribution of genomic alterations in NSCLC is described in prior reports, most have focused on one alteration [3, 4], one histology [5], or a single race [6, 7]. Furthermore, reports describing lung squamous cell carcinoma (LUSC) have emphasized the landscape of squamous-specific alterations, with relatively little attention to targetable mutations in this histologic subset [8]. The prevalence of targetable alterations in less common histologic subtypes of non-small cell lung cancer (i.e., typical and atypical carcinoid tumors, large cell lung cancer) is also not well characterized.

Furthermore, although prior reports have described some differences in mutation profiles based on self-reported race [9], few have explored the correlation between genetically defined ancestry and somatic mutation frequency in detail. In admixed Latin American populations, native American ancestry has been shown to strongly influence the frequencies of *KRAS*, *EGFR*, and *ALK* mutations in NSCLC [10]. Both known and yet-to-be-discovered associations are of interest with respect to screening different populations for such mutations and may uncover mechanistic insight into mutation occurrence.

The American Association for Cancer Research (AACR) launched the Project Genomics Evidence Neoplasia Information Exchange (GENIE), an effort to bring together genomic data from multiple academic and biopharma institutions to enhance the sharing of clinical and genomic information. Here, we utilized the GENIE resource to examine the distribution of targetable genomic alterations in 8 genes in a cohort of 8675 patients with NSCLC and highlight differences across histology, sex, and smoking status. Recognizing the known limitations of self-reported race, we also developed a genetic ancestry inference method to determine ancestry components in each subject quantitatively and used this information to examine correlations between genomic alterations in these 8 genes and quantitative ancestry measurements.

## Methods

### Study design and patient cohorts

We identified patients with solid tumors from the publicly available AACR GENIE 10.1-registry, which is a multi-phase, multi-year, data-sharing project that captures genomic data for cancer patients at multiple institutions (https://www.aacr.org/wp-content/uploads/2021/07/GENIE_10.0-public_data_guide.pdf) [11]. All patients with NSCLC that met the following two criteria were included: (1) tumor sequencing data was available with bait-sets covering single nucleotide variants (SNVs), copy number alterations (CNAs), and fusion variants for 8 genes (*EGFR*, KRAS, *ALK*, *MET*, *ROS1*, *BRAF*, *RET*, and *NTRK*) and (2) gender and race were available. A total of 8675 patients met the inclusion criteria and were separated into a discovery cohort (Dana-Farber Cancer Center, DFCI: *N* = 3115) and a validation cohort (total, *N* = 5560; Duke Cancer Center, DCC: *N* = 5; Memorial Sloan Kettering Cancer Center, MSKCC: *N* = 5405; Vanderbilt-ingram cancer center, VICC: *N* = 150, Additional file 1: Fig. S1). For the DFCI cohort (*n* = 3115 patients**)**, the number of smoking pack-years was determined by chart review, and genetic ancestry was inferred (see the “Estimation of genetic ancestry indices” section). For the validation cohort (*n* = 5560 patients), smoking history was not available, and raw sequencing data was also not available, so self-reported race was utilized.

To minimize the impact of referral bias for rare mutation subsets, and the use of repeat biopsies to assess mechanisms of resistance in patients with driver mutations, we restricted our analysis to the first tumor sample sequenced. For the DFCI cohort, a chart review was used to identify the first tumor sequencing analysis for each patient. For the validation cohort (DCC, MSKCC, VICC), we used tumor sequencing data obtained at the youngest age for each subject.

### Patient consent

DFCI samples were selected and sequenced from patients who were consented under institutional review board (IRB)-approved protocol 11-104 and 17-000 from the Dana-Farber/Partners Cancer Care Office for the Protection of Research Subjects. Written informed consent was obtained from participants prior to inclusion in this study. Secondary analyses of previously collected data were performed with approval from the Dana-Farber IRB: DFCI IRB protocol 18-439 and 19-025; waiver of Health Insurance Portability and Accountability Act (HIPAA) authorization approved for both protocols.

### Tumor specimens

Histologic subtypes included in both the discovery and validation cohorts were lung adenocarcinoma (LUAD), lung adenosquamous carcinoma (LUASC), large cell lung carcinoma (LCLC), lung squamous cell carcinoma (LUSC), and lung carcinoid. Histologic classification was based on the clinical diagnosis rendered in the pathology record for each case. All samples undergoing Oncopanel testing in the DFCI cohort had an official pathology review and formal diagnostic report. Diagnoses were made by board-certified surgical pathologists with subspecialty expertise in pulmonary pathology.

### Genomic analysis

Details of the tissue collection, DNA extraction, and tumor-targeted sequencing were previously described in detail for each of the DFCI (Oncopanel/PROFILE), DCC (Foundation Medicine), MSKCC (MSK-IMPACT), and VICC (Foundation Medicine) cohorts [11–13]. Among the tumor sequencing panels at the four institutions, only bait-sets covering SNVs, CNAs, and fusion variants for all 8 genes were included (DFCI-ONCOPANEL 1-3, DUKE-F1-T5A, VICC-01-T5A, VICC-01-T7, MSK-IMPACT 410, and MSK-IMPACT 468). We focused our mutational analyses on the following alterations: L858R mutations, exon 19 deletions and exon 20 insertions in *EGFR*, *KRAS*^*G12C*^ mutations, exon 14 skipping mutations and amplifications in *MET*, V600E mutations in *BRAF*, and gene fusions in *ALK*, *ROS1*, *RET*, and *NTRK.*

### Next-generation sequencing assays

Specifics about genomic profiling at each center are provided in the AACR Project GENIE Data Guide: https://www.aacr.org/wp-content/uploads/2020/07/20200706_GENIE_10.0-public_data_guide.pdf [11].

### Estimation of genetic ancestry indices

For the DFCI discovery cohort, genetic ancestry was inferred using common polymorphisms called from off-target and on-target sequencing reads [14, 15]. Germline variant imputation was performed across all samples using the STITCH imputation software [16]. This method utilizes ultra-low coverage read data together with the 1000 Genomes reference panel to infer probabilistic germline calls for the autosomal chromosomes. Analysis was restricted to variants with imputation INFO > 0.4 and variant allele frequency (VAF) > 0.01. Ancestry components were inferred for each individual by linear projection using publicly available weights computed by the SNPWEIGHTS software [17], which had been trained on European, West African, and East Asian individuals in the 1000 Genomes project [18] as well as Ashkenazi Jewish Europeans from four large genome-wide association studies [19]. The projection was performed using the imputed dosages and the PLINK2 “--score” function. Ancestry components can have arbitrary rotation/scaling. For visualization purposes, a linear rescaling was applied to the two continental indices such that individuals self-reported as White had a mean score of 0% and individuals self-reported as Asian/Black had a mean score of 100% for the East Asian/African ancestry components, respectively. We note that this is a linear rescaling that does not impact the statistical significance of any association with the indices. As expected, this ancestry score was significantly correlated with self-reported race (Pearson correlation = 0.90; *p* < 2 × 10^−16^) for individuals reporting as either “White/Caucasian,” “Black/African American,” or “Asian” (Additional file 1: Fig. S2). In the DFCI cohort, a European group was defined as individuals with recalibrated African and Asian indices of less than 20% and 15%, respectively, computed by taking 4 standard deviations from the mean for the self-reported White individuals (recognizing that any binarization of quantitative ancestry will be heuristic). For individuals with self-reported race, this European group was >99% self-reported White, and the remaining non-European individuals were 20% self-reported White. Beyond the high correlations with self-reported features, the ancestry score is expected to capture additional variance in genetic ancestry due to admixture and is not susceptible to data missingness or misreporting. We note that we did not separately model Hispanic individuals, which derive ancestry from European, African, and Native American/East Asian populations and thus do not have a single quantitative ancestry score [20]. A Hispanic individual would thus typically have a high East Asian ancestry score (a proxy for Native American ancestry) [21] as well a non-zero African ancestry score, and their indices would be tested separately. However, the number of self-reported Hispanic individuals in this study was very low (1.6%) and thus was not expected to substantially influence the results.

The Ashkenazi Jewish ancestry inference was validated using a previously reported cohort from DFCI of 833 samples that were genotyped on a germline Illumina MEGA SNP array from blood and imputed to the 1000 Genomes reference panel [22]. Ashkenazi Jewish ancestry scores, determined as above from Oncopanel data, were found to be highly concordant with the MEGA SNP AJ genotyping analysis, with a Pearson correlation of 0.99 between the two scores (Additional file 1: Fig. S3). Similarly, high concordance was seen when transforming the SNP-based score into a binary classification for Ashkenazi individuals outside the main distribution of Europeans (87 individuals): the tumor-based score achieved an AUC of 1.0. We also confirmed that individuals self-reporting as religiously Jewish were highly enriched for having a high Ashkenazi Jewish score (Pearson correlation = 0.65, *p* < 10^−100^), though we note that these are not expected to be perfect surrogates. Both the SNP-based score and the tumor-based score achieved an AUC of 0.94 for classifying self-reported religious Jewish status. Hence, ancestry scores inferred from tumors sequenced by Oncopanel are nearly statistically identical to those inferred from germline SNPs and have comparable consistency with self-reported data.

In the DFCI NSCLC cohort (*n* = 3115 patients), patients did not report Ashkenazi Jewish ethnicity, but similar to the test cohort, the AJ ancestry score was significantly associated with self-reported Jewish religion (Pearson correlation = 0.71; *p* < 2 × 10^−16^), demonstrating face validity (though we stress that religion should not be used as a surrogate for ethnicity or genetic ancestry). The Ashkenazi Jewish group was defined as individuals with a rescaled Ashkenazi Jewish score of more than 0.75.

### Statistical analysis

Logistic regression models were used to examine the association between ancestral indices and somatic alterations, with age at diagnosis, pack-years of smoking, tumor histology, and sex used as covariates. The models were used to calculate the odds ratios, 95% confidence intervals (CI), and *p-*values. We applied false discovery rate (FDR) correction by the Bonferroni method for the number of independent tests conducted (significant *q*-value cutoff of < 0.1).

When comparing the frequencies of targetable alterations across different smoking groups (never smokers, 1–15 pack-years, 16–30 pack-years, 31–45 pack-years, 46–59 pack-years, and 60+ pack-years), pairwise Fisher exact tests were performed to calculate odds ratios, 95% confidence intervals (CI), and *p-*values. A logistic regression model was used when pack-years of smoking was used as a continuous variable. Bonferroni correction of *p*-values was applied to account for comparisons among multiple groups. Unless otherwise indicated, only *p*-values that were significant after controlling for false discovery rate are reported (FDR < 0.1).

## Results

### Distribution of targetable genomic alterations across NSCLC histotypes

**Fig. 1:**
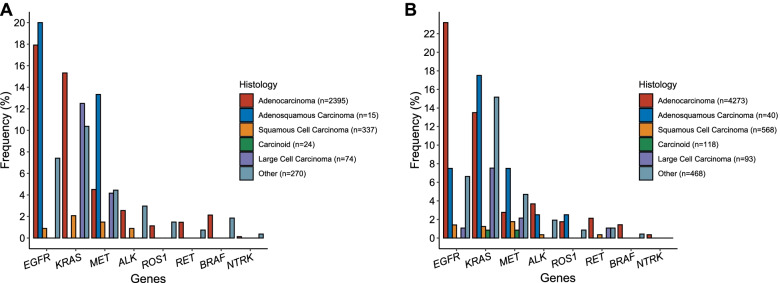
Histology-specific differences in genomic alterations in NSCLC in the discovery (**A**) and validation (**B**) cohorts. Frequency of eight targetable genomic alterations in five main NSCLC histologic subtypes

Among 3115 patients with NSCLC seen at the Dana-Farber Cancer Institute who had tumor sequencing performed (see the “Methods” section, Additional file 2: Table S1), the most common tumor histology was LUAD (2395/3115, 76.9%), followed by LUSC (337/3115, 10.8%) and carcinoid (74/3115, 2.4%). Most of the samples sequenced were derived from the primary cancer site (1863, 59.8%, Table 1 and Additional file 2: Table S1). Targetable genomic alterations in the 8 genes (*EGFR*, *KRAS*, *ALK*, *MET*, *ROS1*, *BRAF*, *RET*, and *NTRK*; see the “Methods” section) were detected in 44.0% of patients with LUAD (1051/3115, Additional file 2: Tables S2-S9), 33% (5/15) of patients with LUASC carcinoma, 17% (4/24) of patients with LCLC, 5.3% (18/337) of patients with LUSC, and 0% (0/74) of patients with lung carcinoid tumors (*n* = 50 typical and *n* = 24 atypical, Fig. 1A). LCLC (*n* = 24) harbored *KRAS*^*G12C*^ mutations at a frequency comparable to LUAD (13% vs. 15.1%, *q-*value = 1), but had a significantly lower frequency of *EGFR* L858R, exon 19 deletions, or exon 20 insertions (0% vs 17.9%, OR = 0 [95% CI = 0–0.76], *p-*value = 0.016). *ALK*/*ROS1*/*NTRK*/*RET* fusions and *BRAF*^*V600E*^ mutations were not detected in any LCLC sample, although the number of samples was small. Targetable alterations in *EGFR*, *KRAS*, *MET*, *ALK*, and *RET* were identified in LUSC samples, although at much lower frequency compared to LUAD (all *q-*values < 0.05). *ALK*, *ROS1*, and *NTRK* gene fusions were found almost exclusively in patients with LUAD. The validation cohort of 5560 GENIE patients from several institutions showed a similar distribution of mutations for each histology (Additional file 2: Table S10, Fig. 1B), with the exception that mutation frequencies in rare pathologic subtypes (adenosquamous, large cell) were more variable, as expected.

**Table 1:** Baseline characteristics and frequency of eight targetable genomic alterations of 3115 NSCLC patients from the DFCI cohort according to tumor histology

DFCI cohort		Adenocarcinoma	Squamous cell carcinoma	Lung carcinoid	Large cell carcinoma	Adenosquamous carcinoma	Other^a^	Total
Age at diagnosis	Median	66	68	58.5	64	67	65.5	66
Sex	Male (%)	883 (36.9%)	204 (60.5%)	16 (22%)	10 (42%)	8 (53%)	135 (50%)	1256 (40.4%)
	Female (%)	1512 (63.1%)	133 (39.5%)	58 (79%)	14 (58%)	7 (47%)	135 (50%)	1859 (59.6%)
Site	Primary (%)	1400 (58.5%)	244 (72.4%)	62 (84%)	13 (54%)	14 (93%)	133 (49.3%)	1866 (59.9%)
	Metastasis (%)	910 (38.0%)	77 (22.8%)	11 (15%)	11 (46%)	1 (7%)	111 (41.1%)	1121 (36.0%)
	Local Recurrence (%)	36 (1.5%)	9 (2.7%)	0 (0%)	0 (0%)	0 (0%)	5 (1.9%)	48 (1.5%)
	Unspecified (%)	49 (2.0%)	7 (2.1%)	1 (1%)	0 (0%)	0 (0%)	21 (7.8%)	80 (2.6%)
Smoking status	Current (%)	375 (15.7%)	91 (27.0%)	3 (4%)	8 (33%)	2 (13%)	52 (19.3%)	531 (17.0%)
	Former (%)	1420 (59.3%)	214 (63.5%)	36 (49%)	12 (50%)	11 (74%)	170 (63.0%)	1863 (59.8%)
	Never (%)	600 (25.0%)	32 (9.4%)	35 (48%)	4 (16%)	2 (13%)	48 (17.8%)	721 (23.1%)
Targetable alterations	*EGFR*^*L858R*/*exon19*^^*del*^^/*exon20ins*^ (%)	429 (17.9%)	3 (0.9%)	0 (0%)	0 (0%)	3 (20%)	20 (7.4%)	455 (14.6%)
	*KRAS*^*G12C*^ (%)	367 (15.3%)	7 (2.1%)	0 (0%)	3 (12%)	0 (0%)	28 (10.4%)	405 (13.0%)
	*MET* ^*amp/exon14*^^*skipping*^ (%)	108 (4.5%)	5 (1.5%)	0 (0%)	1 (4%)	2 (13%)	12 (4.4%)	128 (4.1%)
	*BRAF*^*V600E*^ (%)	51 (2.1%)	0 (0%)	0 (0%)	0 (0%)	0 (0%)	5 (1.9%)	56 (1.8%)
	*ALK* fusion (%)	61 (2.5%)	3 (0.9%)	0 (0%)	0 (0%)	0 (0%)	8 (3.0%)	72 (2.3%)
	*ROS1* fusion (%)	27 (1.1%)	0 (0%)	0 (0%)	0 (0%)	0 (0%)	4 (1.5%)	31 (1%)
	*RET* fusion (%)	35 (1.5%)	0 (0%)	0 (0%)	0 (0%)	0 (0%)	2 (0.7%)	37 (1.2%)
	*NTRK* fusion (%)	3 (0.1%)	0 (0%)	0 (0%)	0 (0%)	0 (0%)	1 (0.4%)	4 (0.1%)
Total	*N* (% of total samples)	2395 (76.9%)	337 (10.8%)	74 (2.4%)	24 (0.8%)	15 (0.5%)	270 (8.6%)	3115

^a^ Includes non-small cell lung cancer not otherwise specified (NOS), pleomorphic carcinoma of the lung, neuroendocrine lung carcinoma NOS, sarcomatoid carcinoma of the lung, adenoid cystic carcinoma of the lung, giant cell carcinoma of the lung, spindle cell carcinoma of the lung, inflammatory myofibroblastic lung tumor, basaloid carcinoma of the lung, ciliated muconodular papillary tumor of the lung, and lymphoepithelioma-like carcinoma of the lung

### Smoking status and targetable alterations in patients with lung adenocarcinoma and squamous cell carcinoma

**Fig. 2:**
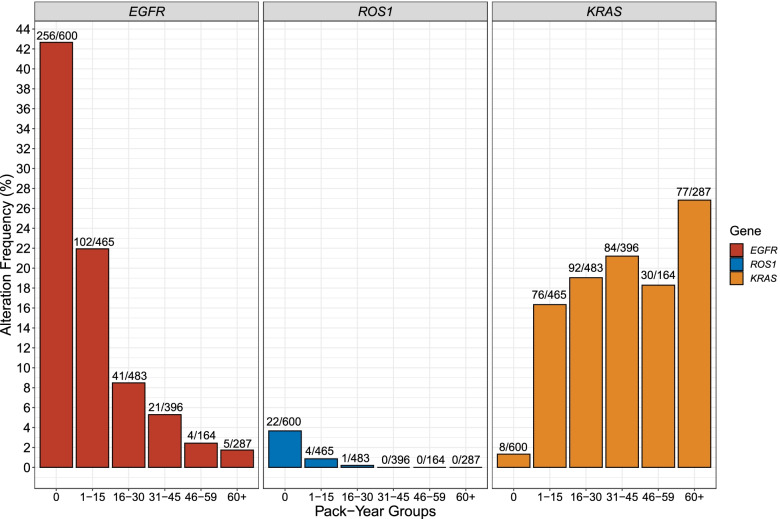
*EGFR* ^*L858R/ex19del/ex20ins*^, *ROS1 *fusions, and *KRAS*^*G12C*^ in lung adenocarcinoma, grouped according to pack-years of smoking

To assess the effect of smoking on the frequency of each of the eight targetable alterations, we limited our analysis to patients with LUAD treated at DFCI with available quantitative smoking history data (Additional file 1: Fig. S4). To examine whether there is an incremental dose-effect of pack-years on the mutation rate, the cohort was split into six groups: never smokers, 1–15 pack-years, 16–30 pack-years, 31–45 pack-years, 46–60 pack-years, and 60+ pack-years, and the frequency of alterations was determined for each group (Additional file 2: Table S11). The prevalence of *EGFR* L858R, exon 19 deletions, and exon 20 insertions steadily declined with increasing smoking exposure (Fig. 2). *ROS1* fusion mutations also declined dramatically with smoking exposure such that only one LUAD patient with 16 or more pack-years had a ROS1 fusion (1/1330, 0.07%, Fig. 2). In contrast, and as expected, *KRAS*^*G12C*^ mutation prevalence in our cohort showed an opposite pattern, with a fifteen-fold increase in the lightest smoking group, 1–15 pack-years (OR = 15 [7–35]; Bonferroni-corrected *p*-value < e−16, Fig. 2) compared to never smokers, in whom *KRAS*^*G12C*^ mutations were rare, seen in 8 of 600 (1.4%). In addition, the frequency of *KRAS*^*G12C*^ mutation was relatively stable over all smoking groups, from 1–15 pack-years to 60+ pack-years (Fig. 2). Note that we do not interpret these findings as indicating that smoking is protective against *EGFR* and other targetable mutations; rather smoking carcinogen-driven mechanisms of tumorigenesis steadily increase with increasing smoke exposure, causing an increase in *KRAS* mutations, and non-targetable mutations, and a relative decline in the frequency of other targetable driver mutations in the smoking-exposed population.

*ALK* fusion frequency considerably decreased when comparing never smokers to 16+ pack-year smokers (Bonferroni-corrected *p*-values < 0.05, Additional file 1: Fig. S4). Consistent with previous studies, there was no significant association between *MET* and *BRAF* alteration prevalence and smoking exposure (*q-*values > 0.1, Additional file 1: Fig. S4, Additional file 2: Table S12). Associations with *ALK* fusions, KRAS^*G12C*^ mutations, and *ROS1* fusions were maintained when analyzing smoking pack-years as a continuous variable (Additional file 2: Table S12). *RET* and *NTRK* fusions were too few to permit a pack-year association analysis.

For the subset of patients with LUSC, there was dramatic enrichment for “LUAD-specific” targetable alterations in the never-smoking group. Targetable alterations were seen in 6 of 32 (19%) never smokers with LUSC, in comparison to 12 of 305 (3.9%, *p* = 0.003) among smokers with LUSC. The 6 alterations seen in never smokers were 2 *EGFR* and 4 *MET* mutations. Seven of 12 (58%) alterations in smoker squamous cell carcinoma patients were *KRAS*^*G12C*^ (Additional file 2: Table S13).

### Targetable alterations across continental genetic ancestries and self-reported race

To compare the somatic profiles of targetable alterations in individuals from different ancestral populations, we use a novel method to calculate ancestry indices for all DFCI subjects in the discovery cohort including African, Ashkenazi Jewish, and Asian markers [15] (see the “Methods” section). The ancestry score was significantly associated with self-reported ancestry (Additional file 1: Fig. S3) for individuals reporting as either “White/Caucasian,” “Black/African American,” or “Asian”. However, these ancestry indices provided quantitative information on admixture, which was present in many patients.

**Fig. 3:**
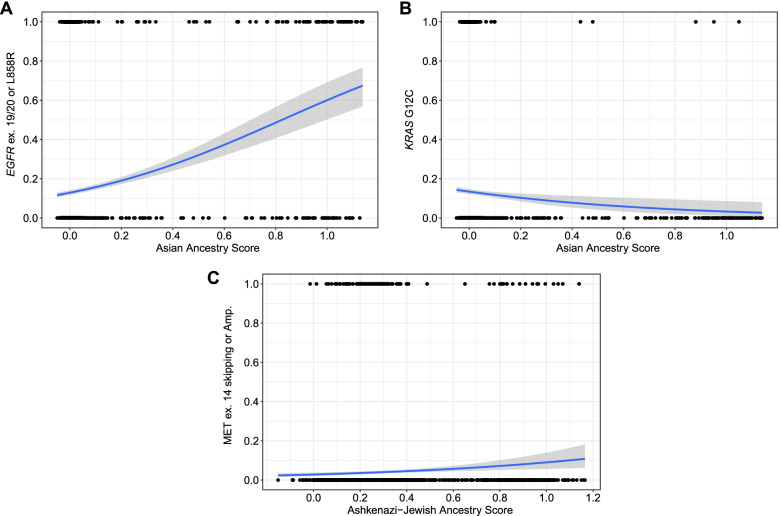
Logistic regression plots showing the association between continuous ancestry scores and the presence (1) or absence (0) of targetable alterations. **A** *EGFR* ^L858R/ex19del/ex20ins^ and Asian ancestry (AA) score. **B** *KRAS*^*G12C*^ and Asian Ancestry (AA) score. **C** *MET*^*amp/ex14skipping*^ and Ashkenazi Jewish ancestry (AJ) score. Each dot represents an individual patient

For each of the eight targetable alterations, we performed continental ancestry-specific multivariate logistic regression in the DFCI cohort, adjusting for age at diagnosis, tumor histology, number of smoking pack-years, and sex, using European ancestry individuals as the reference (see the “Methods” section). Consistent with previous reports [23], *EGFR* mutations were highly enriched in patients with higher Asian ancestry score (OR = 4.9, 95% CI = 3.1–7.9, *p* < 0.0001, *q-*value < 0.1; Fig. 3A, Additional file 2: Table S14). Conversely, *KRAS*^*G12C*^ mutations were much less frequent in patients with high Asian ancestry, again while controlling for age, histology, smoking, and sex (Fig. 3B, Additional file 2: Table S14). Limiting to *EGFR*-wild type NSCLC, patients of Asian ancestry still harbored significantly less *KRAS*^*G12C*^ mutations compared to other continental genetic ancestries (Additional file 2: Table S15). None of the remaining six targetable alterations was significantly enriched in any of the continental ancestries after accounting for sex differences and smoking history (Additional file 2: Table S14).

Among the validation cohort of 5560 samples, self-reported Asians were again enriched for *EGFR* mutations compared to Whites and Blacks (Asians: 267/538, 49.6% vs Blacks: 69/309, 22.3% vs Whites: 676/4591, 14.7%, *p* < 0.0001). In contrast, *KRAS*^*G12C*^ mutations were depleted in Asians (Asians: 18/538, 3.3% vs Blacks: 28/309, 9.1% vs Whites: 616/4591, 13.4%, *p*<0.0001, Additional file 2: Table S16).

### Targetable alterations and tumor mutational burden in patients of Ashkenazi Jewish ancestry

Within the European cohort (*n* = 2837 patients, see the “Methods” section), we also examined the potential association between Ashkenazi Jewish (AJ) ancestry and mutation frequency. Patients with high AJ ancestry had a lower median TMB (6.1 mutations/MB, *n* = 215) compared to non-Ashkenazi Europeans (9.6 mutations/MB, *n* = 2622, *p* < 0.0001, Additional file 1: Fig. S5), an association which persisted after correcting for pack-years of smoking as a continuous variable (*p* < 0.0001). For validation, genetic ancestry inference was performed on tumor/normal paired samples from The Cancer Genome Atlas (TCGA) LUSC (*n* = 429) and LUAD (*n* = 441) cohorts. In the TCGA LUAD and LUSC cohorts, patients with high AJ ancestry had lower median TMB compared to non-AJ Europeans (Median TMB (LUAD and LUSC AJ) = 5.0 vs median TMB (LUAD and LUSC non-AJ) = 6.7, *p* > 0.05, Additional file 1: Fig. S6, Additional file 2: Table S17). This was not statistically significant, likely due to the small numbers of AJ samples (*n* = 24 total for LUAD and LUSC combined) but had the same trend.

Targetable alterations in three genes were found in at least 5% of patients with high AJ ancestry: *EGFR*, *KRAS*, and *MET*. Strikingly, patients with high AJ ancestry were significantly more likely to harbor *MET* exon 14 skipping mutations and amplifications (OR = 2.1; 95% CI = 1.0–4.3, *p* = 0.039; Fig. 3C) after accounting for age at diagnosis, tumor histology, number of pack-years, and sex. Of 215 patients with high AJ ancestry, 20 (9.3%) harbored *MET* genomic alterations compared to 97 of 2622 (3.7%) of Europeans with low AJ ancestry (*p* = 0.0004). Other targetable alterations were not significantly associated with AJ ancestry (Additional file 2: Table S18).

**Fig. 4:**
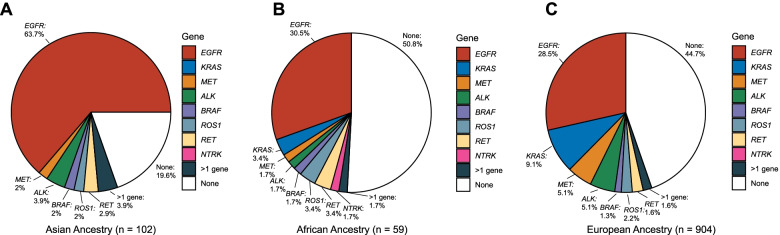
Distribution of targetable alterations by inferred ancestral groups among light smokers (**≤**15 pack-year smoking history) with LUAD

We pooled subjects with either never or light smoking history (1–15 pack-years) and adenocarcinoma histology and calculated the frequency of targetable alterations for each continental ancestral population. Asians harbored significantly more targetable alterations (82/102, 80%) compared to Africans (29/59, 49%) or Europeans (509/904, 56%, *p* < 0.001, Fig. 4), and these differences were mainly due to differences in *EGFR* mutation frequency (64%, 30%, 28%, respectively). There was no difference between Ashkenazi and non-Ashkenazi Europeans (51% vs. 49%, respectively).

## Discussion

It is well known [10, 24, 25] that genetic ancestry influences the risk of cancer gene mutation, both through strong single gene (Mendelian) mechanisms and through collective weaker association effects. Genetic ancestry can be assessed quantitatively as done here and provide a more direct and accurate means of inferring population ancestry than questionnaire information [25, 26].

We computed ancestry indices for Asian, African, and Ashkenazi Jewish components for 3115 patients with NSCLC sequenced at DFCI, enabling us to assess the potential influence of these genetic backgrounds on mutation development. We confirmed enrichment of *EGFR* L858R mutations, exon 19 deletions, and exon 20 insertions, and depletion of *KRAS*^*G12C*^ mutations in patients with higher Asian ancestry. Associations between Asian ancestry and gene fusions (*ALK*, *ROS1*, *RET*, and *NTRK*) were found but were strongly confounded by smoking habits*.* Interestingly, among never and light smokers, 80% of those with high Asian ancestry harbored targetable alterations (Fig. 4), much higher than what was seen in our European ancestry and African ancestry patients. This suggests that, with the current armamentarium of targeted therapies in NSCLC, patients with higher Asian ancestry may benefit from a wider array of treatment options.

The AJ population is known to be enriched for several rare germline variants in genes that predispose to cancer development, including *BRCA1* and *BRCA2*. Furthermore, recently, a rare variant in *ATM* (11-108326169-C-T (GRCh38), p.Leu2307Phe) present in AJ populations at an allele frequency (AF) of 0.029, and in all other populations at AF < 0.0003 (https://gnomad.broadinstitute.org/variant/11-108326169-C-T?dataset=gnomad_r3), was found to confer a marked relative risk (OR 3–8) of development of LUAD [27]. We observed that patients with a higher AJ ancestry score were more likely to harbor *MET* exon 14 skipping mutations and amplifications (Fig. 3C).

We also showed that the overall prevalence of targetable, traditionally LUAD, mutations in LUSC patients is about 5%, similar to the prior TCGA analysis in which such mutations were seen in 5 of 178 (3%) [8]. In addition, targetable mutations were greatly enriched in the never-smoking subset (OR = 5.2). Targetable LUAD mutations were also found in 17% of patients with large cell lung carcinoma (LCLC). These findings suggest that genetic testing for all patients with LUSC and LCLC is appropriate, similar to recent considerations [28]. None of the 143 carcinoid lung tumors (typical and atypical) that were sequenced had any LUAD targetable alterations, as expected. The statistical power of our observations reflects the value of large aggregate data resources such as GENIE.

Our analysis of the effect of smoking on targetable mutation prevalence confirmed some known associations. Moreover, it highlighted novel associations with clinical implications in several respects. Both *MET* and *BRAF* targetable alterations showed a similar mutation frequency independent of smoking exposure (Additional file 1: Fig. S3). This may also be true for *RET* and *NTRK* fusions, but the numbers were too few for statistical confidence. *EGFR*, *ALK*, and *ROS1* alterations declined significantly with increasing smoking exposure. This was most dramatic for *ROS1* fusions which were seen in 1/1330 (0.07%) patients with >16 pack-years exposure, in contrast to a 3.7% frequency in never smokers (Fig. 2). With the absence of *ROS1* fusions detected in heavy smokers in our cohort, immunohistochemistry of *ROS1* might be considered unnecessary in patients with extensive smoking history (>15 pack-years).

Our work has several limitations. First, the cohorts studied were from four (predominantly two) large tertiary academic cancer institutions whose patient populations are likely biased toward patients with unusual clinical features, including young age at onset, and rare targetable mutations. It is likely that this occurrence elevated the frequency of all of the rare cancer variants studied here. However, note that we considered only the first tumor sample sequenced to attempt to overcome this referral bias (see the “Methods” section). Second, targeted panel sequencing was used to identify mutations and copy number events in tumor samples only in the DFCI/DCC/VICC cohorts; this makes it possible that some of the genomic alterations captured in these cohorts are germline rather than somatic. However, germline alterations in the 8 targetable variants assessed are extremely rare [29]. Third, targeted panel DNA sequencing can miss structural variants; RNA-based methods are more sensitive for detection of fusion oncogene-activating events. As such, one might expect reduced detection of fusion variants in this series, meaning that the prevalence of such variants may be even higher than reported here.

## Conclusions

In summary, we use a novel computational approach to infer ancestry quantitatively in patients with NSCLC. This method may be useful in other studies to elucidate the genetic contribution to cancer disparities. Using these ancestry indices, we demonstrate that the prevalence of targetable genomic alterations in NSCLC is variable across different populations after accounting for smoking and other factors. We provide further clarity on the impact of smoking pack-years on the frequency of targetable alterations and characterize the prevalence of these alterations in less common lung cancer histologies. Finally, we show that the Ashkenazi Jewish population appears to have an increased prevalence of *MET* mutations and amplifications in LUAD, which warrants further investigation to validate this observation, explore mechanism, and determine clinical relevance.

## Supplementary Information

**Additional file 1: Fig. S1.** Consort diagram of the analysis workflow. **Fig. S2.** Correlation between genetically-inferred ancestry and self-reported race among NSCLC patients in the DFCI cohort. **Fig. S3.** Ashkenazi ancestry score benchmarking**. Fig. S4.** *MET* amplifications/exon 14 skipping mutations, *BRAF* ^*V600E*^ mutations, *ALK/RET/NTRK* fusions in lung adenocarcinoma, grouped according to pack-years of smoking in the DFCI cohort. **Fig. S5.** Tumor mutational burden in Ashkenazi vs. non-Ashkenazi Jewish Europeans with NSCLC in the DFCI cohort. **Fig. S6.** Tumor mutational burden in Ashkenazi vs. non-Ashkenazi Jewish Europeans with NSCLC in the TCGA.

**Additional file 2: Table S1.** Master Table of clinicopathological characteristics of 3,115 patients with NSCLC sequenced at DFCI. **Table S2.** *EGFR*^*L858R/ex19del/ex20ins*^ variants reported in patients with NSCLC sequenced at DFCI. **Table S3.** *KRAS*^G12C^ variants reported in patients with NSCLC sequenced at DFCI. **Table S4.** Exon 14 skipping mutations and/or amplifications in *MET* reported in patients with NSCLC sequenced at DFCI. **Table S5.** *ALK* fusions reported in patients with NSCLC sequenced at DFCI. **Table S6.** *ROS1* fusions reported in patients with NSCLC sequenced at DFCI. **Table S7.** *BRAF*^*V600E*^ variants reported in patients with NSCLC sequenced at DFCI. **Table S8.** *RET* fusions reported in patients with NSCLC sequenced at DFCI. **Table S9.** *NTRK* fusions reported in patients with NSCLC sequenced at DFCI. **Table S10.** Master Table of clinicopathological characteristics of 5,560 patients with NSCLC sequenced at one of three institutions (DCC, VICC, MSKCC). **Table S11.** Frequency of eight targetable alterations in DFCI LUAD patients by pack-year groups. **Table S12.** Comparison of targetable alteration frequencies in pack-year groups among patients with NSCLC at DFCI. **Table S13.** Distribution of targetable alterations by pack-year smoking history among patients with LUSC at DFCI. **Table S14.** Multivariable logistic regression model of eight targetable alterations, incorporating continental ancestry scores, age at diagnosis, smoking pack-year history, sex and histology DFCI cohort). **Table S15.** Comparison of *KRAS*^*G12C*^ mutation frequencies across continental ancestries (discovery cohort from DFCI) and self-reported racial groups (validation cohort from DCC, MSKCC, and VICC). **Table S16.** Multivariable logistic regression model of eight targetable alterations, incorporating sex and histology (validation cohort). **Table S17.** Tumor mutational burden and Ashkenazi Jewish ancestry assessment in TCGA LUAD and LUSC cohorts. **Table S18.** Multivariable logistic regression model of eight targetable alterations, incorporating Ashkenazi Jewish ancestry scores, age at diagnosis, smoking pack-year history, sex and histology.

## Data Availability

All patient-level data is publicly available on cBioPortal.org (https://genie.cbioportal.org/study/summary?id=genie_public). All additional unidentifiable clinical data is available within the article and its supporting files. The individual-level sequencing and imputation data cannot be made publicly available because the research participant consent does not include authorization to share identifiable data. The full analysis workflow for ancestry imputation is available at https://github.com/gusevlab/panel-imp [15]. A containerized version of the imputation pipeline is available at https://hub.docker.com/r/stefangroha/stitch_gcs.
