# Supplementary material for: Variation in targetable genomic alterations in non-small cell lung cancer by genetic ancestry, sex, smoking history, and histology
Source: Genome Med. 2022 Apr 15;14:39. doi: 10.1186/s13073-022-01041-x (PMC9013075; doi:10.1186/s13073-022-01041-x)
Supplement: Supplementary file 1 — Additional file 1: Fig. S1. Consort diagram of the analysis workflow. Fig. S2. Correlation between genetically-inferred ancestry and self-reported race among NSCLC patients in the DFCI cohort. Fig. S3. Ashkenazi ancestry score benchmarking. Fig. S4. MET amplifications/exon 14 skipping mutations, BRAF V600E mutations, ALK/RET/NTRK fusions in lung adenocarcinoma, grouped according to pack-years of smoking in the DFCI cohort. Fig. S5. Tumor mutational burden in Ashkenazi vs. non-Ashkenazi Jewish Europeans with NSCLC in the DFCI cohort. Fig. S6. Tumor mutational burden in Ashkenazi vs. non-Ashkenazi Jewish Europeans with NSCLC in the TCGA. [file 13073_2022_1041_MOESM1_ESM.pdf]

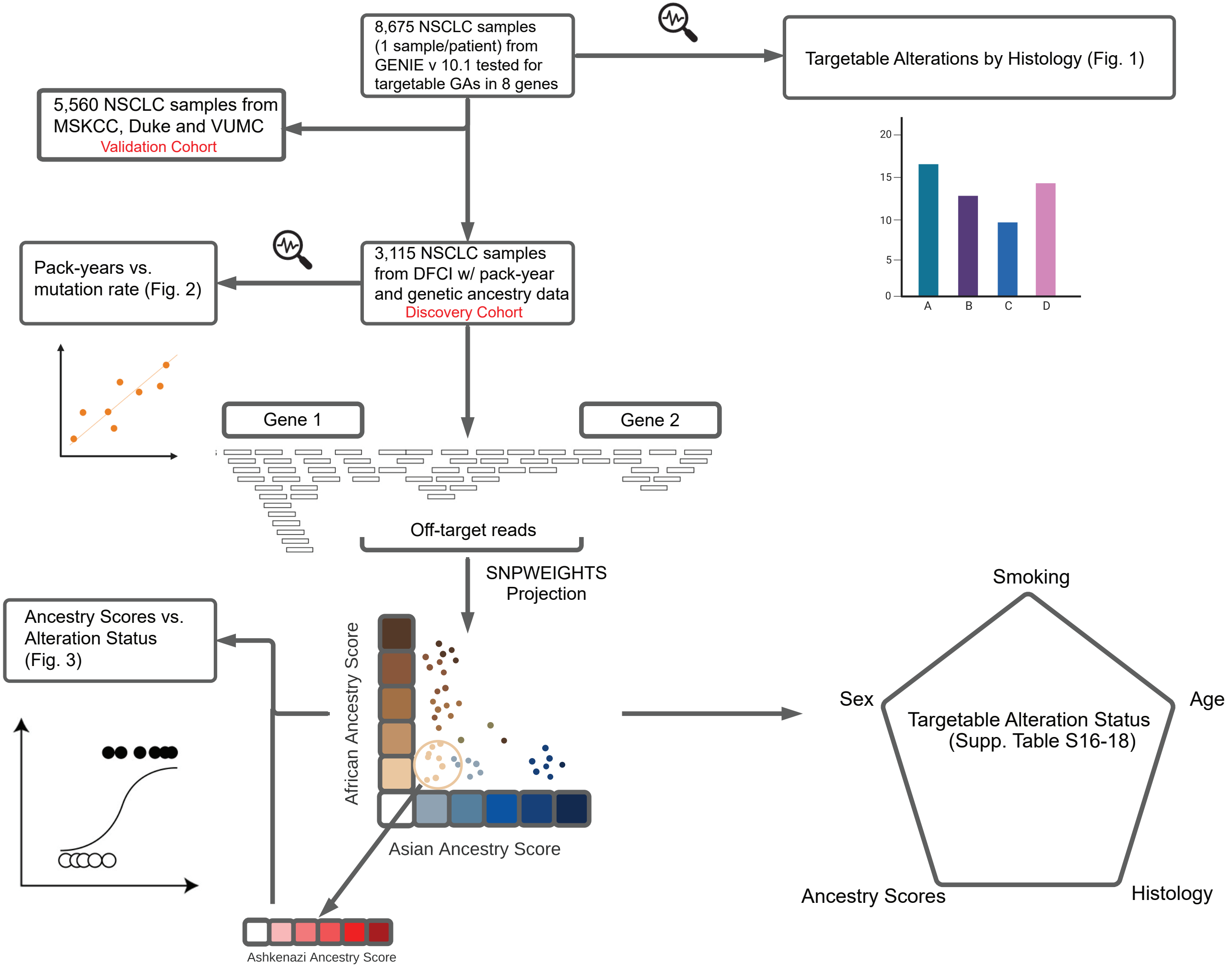

Fig. S1. Consort diagram of the analysis workflow on 8,675 GENIE NSCLC samples.

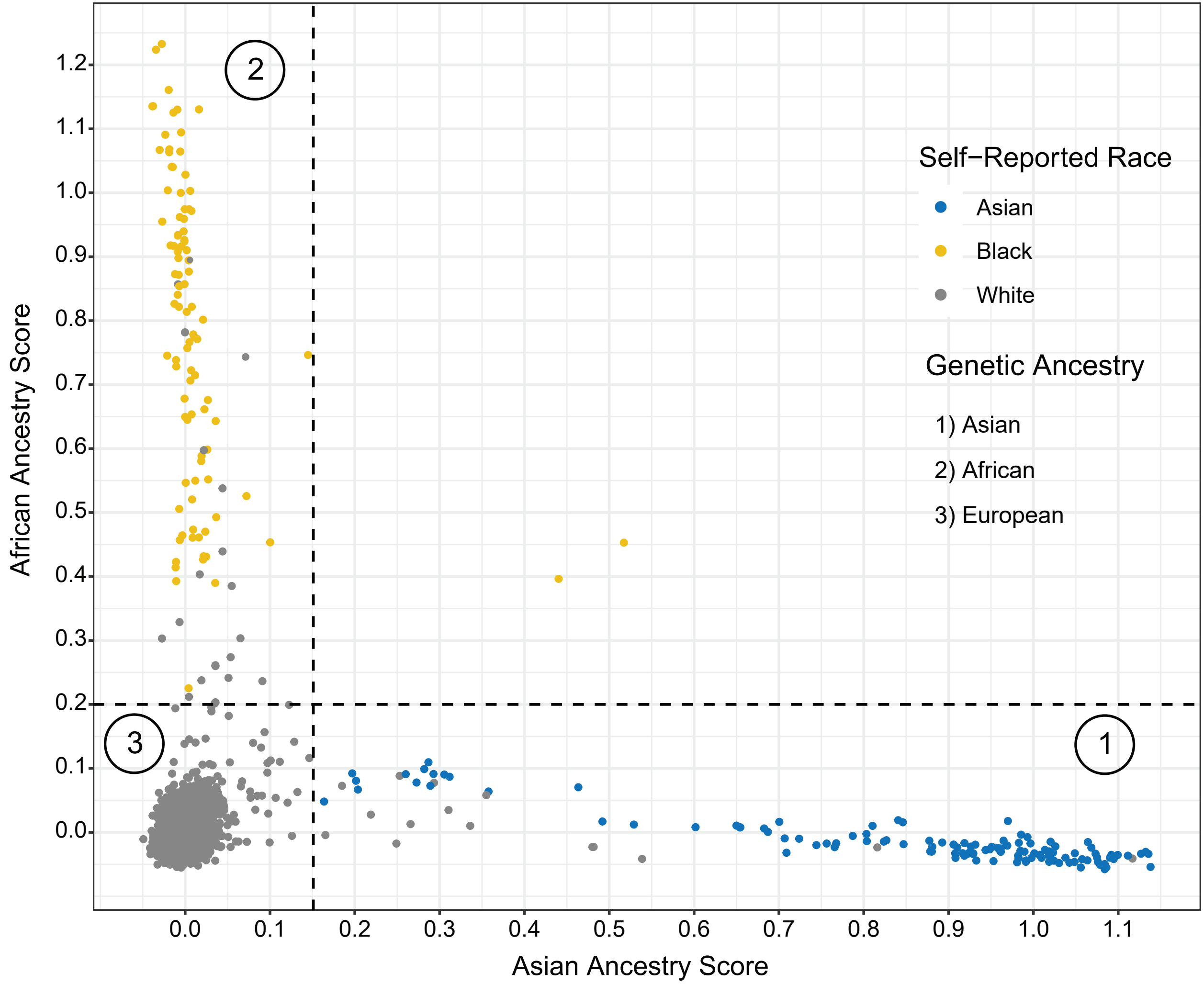

Fig. S2. Correlation between genetically-inferred ancestry and self-reported race among NSCLC patients in the DFCI cohort.

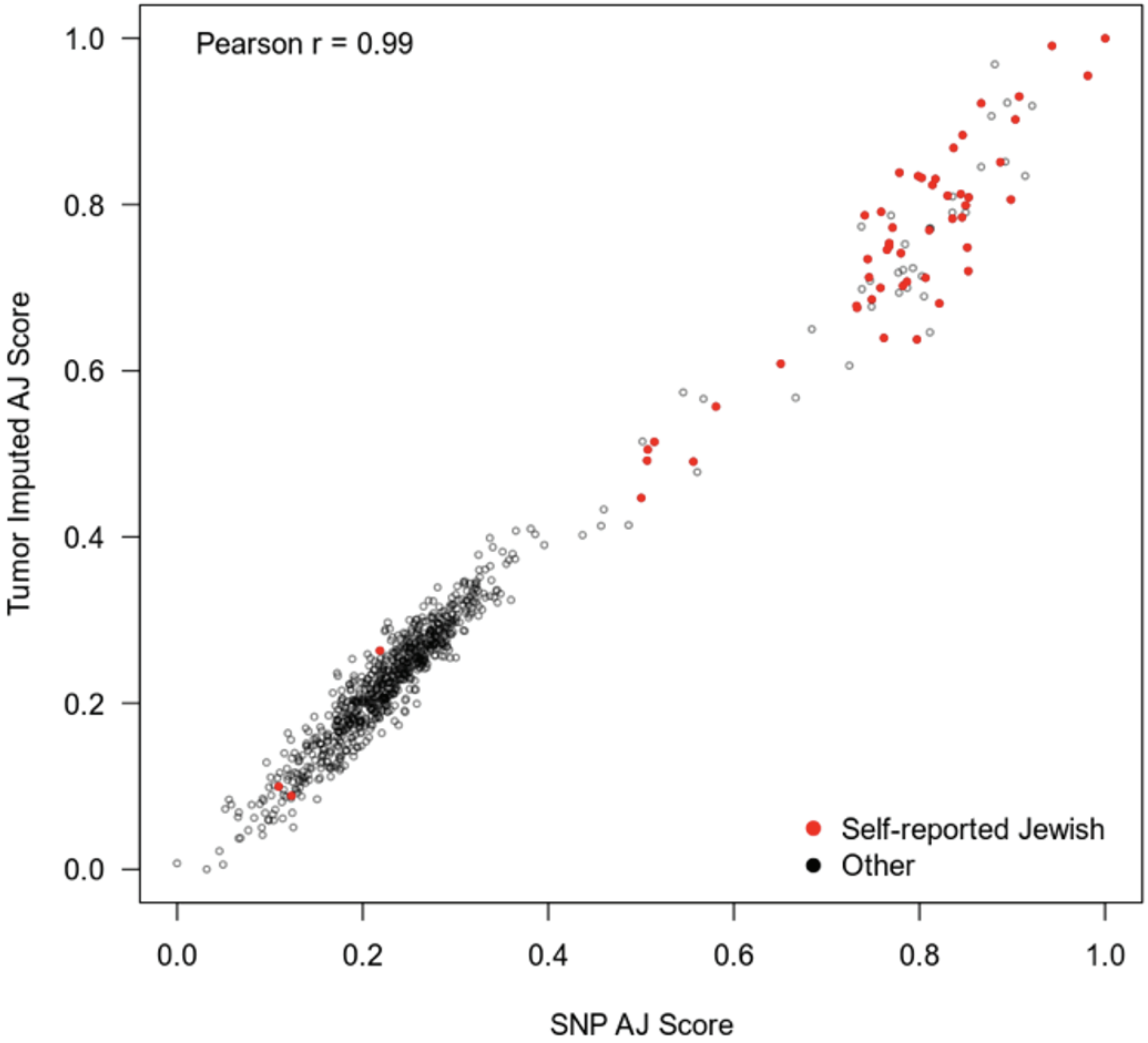

Fig. S3. Ashkenazi ancestry score benchmarking. Scatterplot of the ancestry score inferred from germline SNPs (x-axis) and tumor-based imputation (y-axis) among a test cohort of 833 samples genotyped on a germline Illumina MEGA SNP array from blood and imputed to the 1000 Genomes reference panel. Each point represents an individual, withindividuals self-reporting as religiously Jewish shown in red.

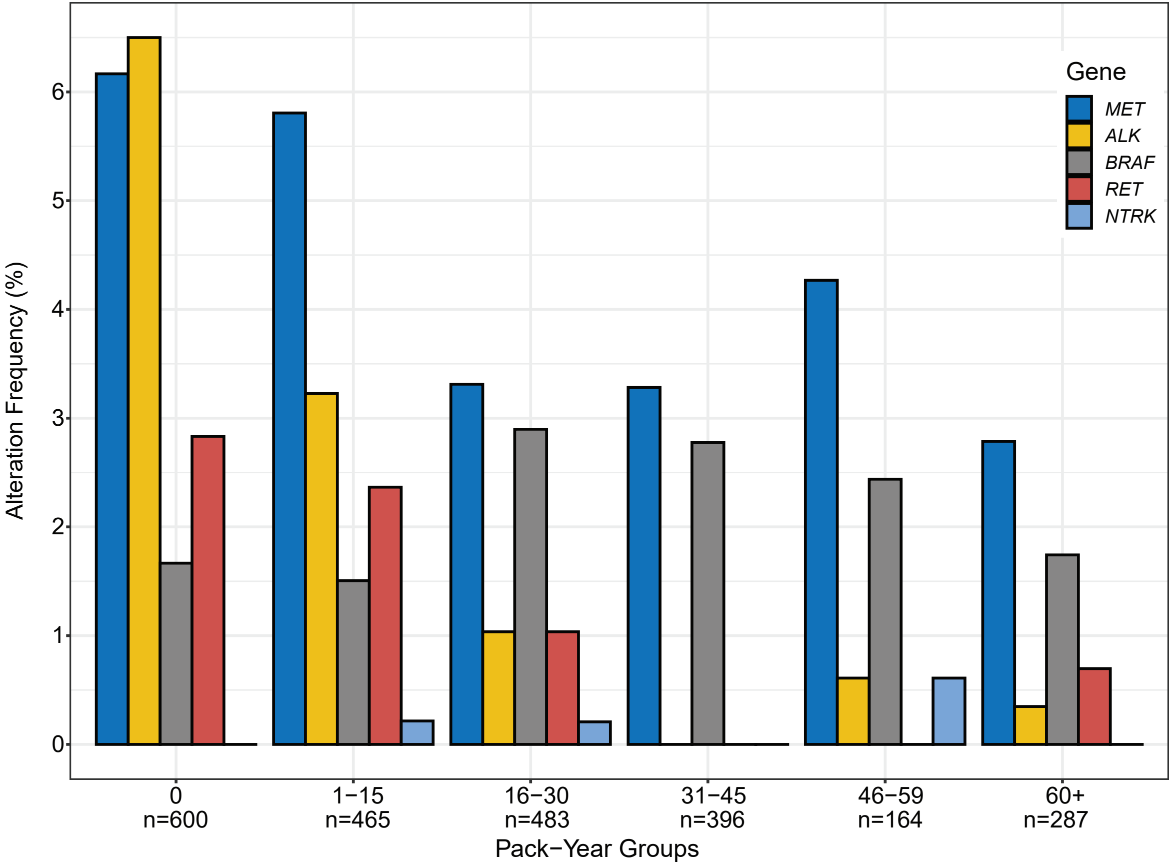

Fig. S4. *MET* amplifications/exon 14 skipping mutations, *BRAF* V600E mutations, *ALK/RET/NTRK* fusions in lung adenocarcinoma, grouped according to pack-years of smoking in the DFCI cohort.

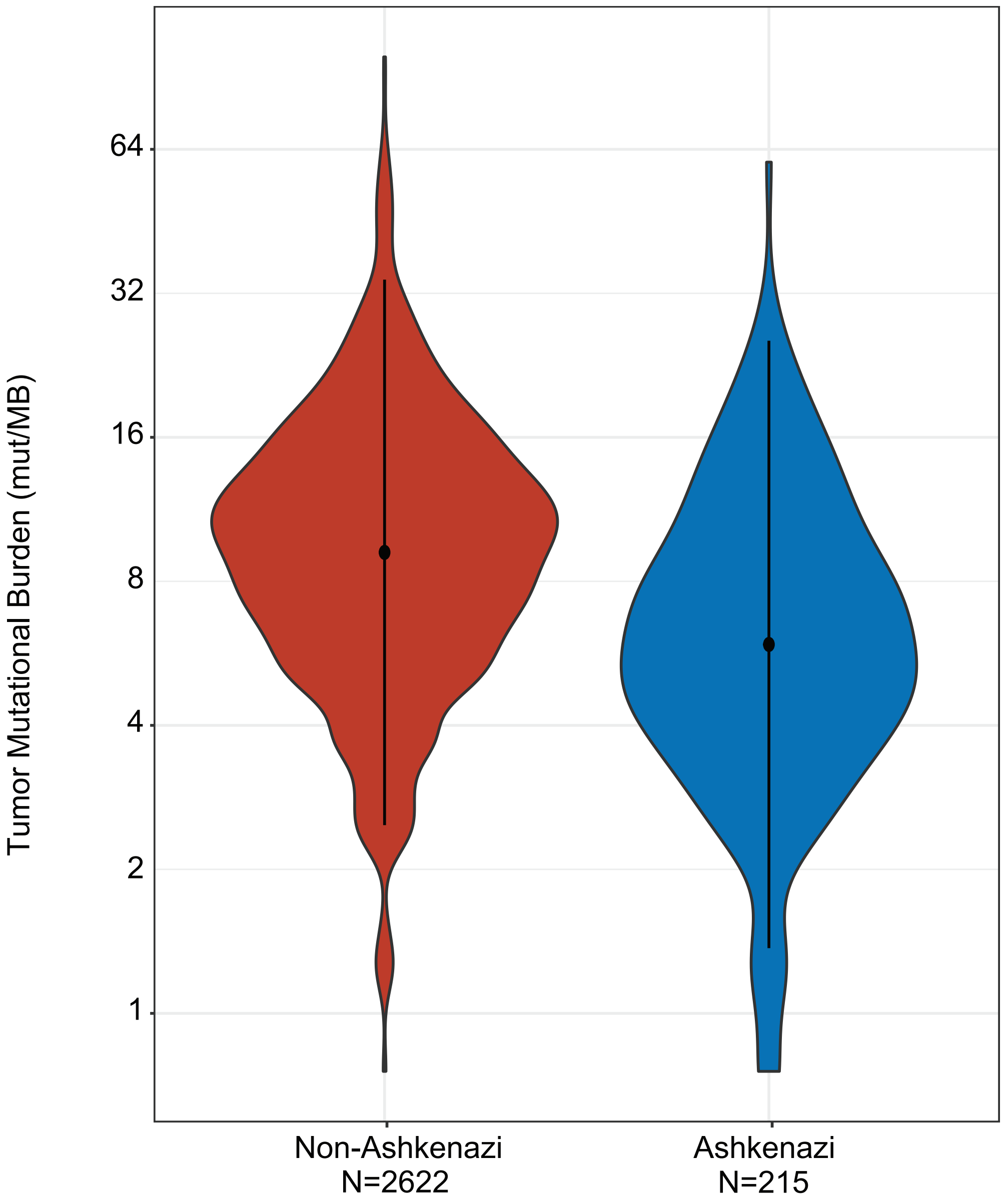

Fig. S5. Tumor mutational burden in Ashkenazi vs. non-Ashkenazi Jewish Europeans with NSCLC in the DFCI cohort. Each dot represents an individual patient.

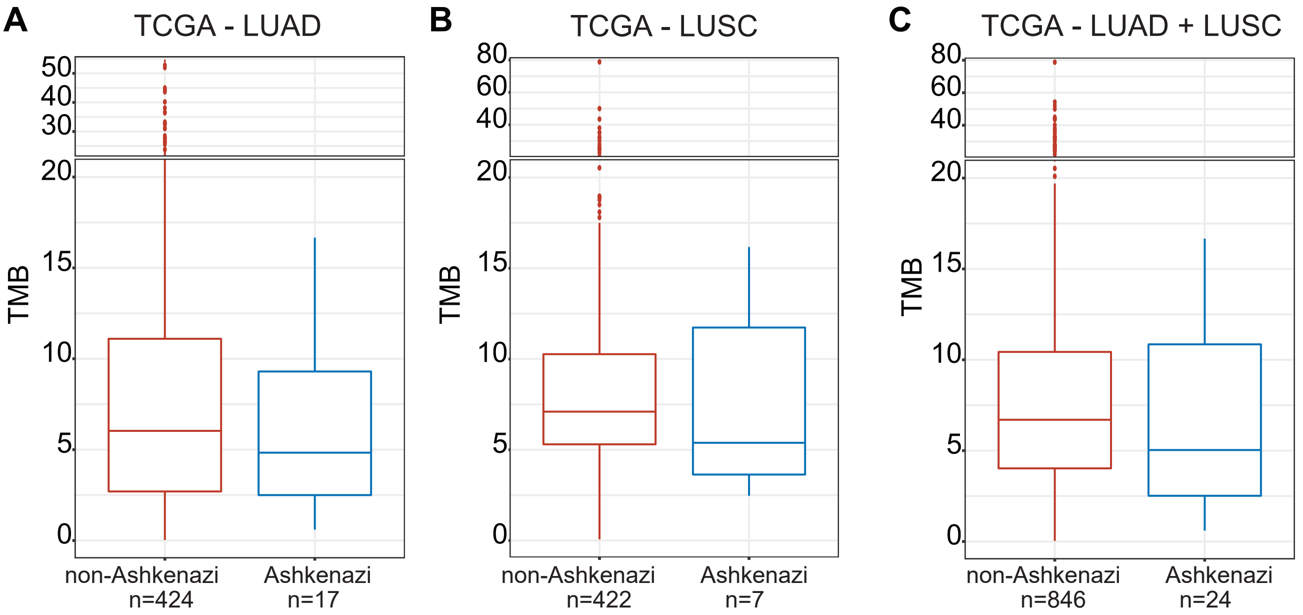

Fig. S6. Tumor mutational burden in Ashkenazi vs. non-Ashkenazi Jewish Europeans with NSCLC in the TCGA LUAD (A), LUSC (B) and combined (C) cohorts.
